# Supplementary material for: Lamin B1 is required for mature neuron-specific gene expression during olfactory sensory neuron differentiation
Source: Nat Commun. 2017 Apr 20;8:15098. doi: 10.1038/ncomms15098 (PMC5411488; doi:10.1038/ncomms15098)
Supplement: Supplementary Information — Supplementary Figures, Supplementary Table, Supplementary Methods and Supplementary References. [file ncomms15098-s1.pdf]

Supplementary Figure 1

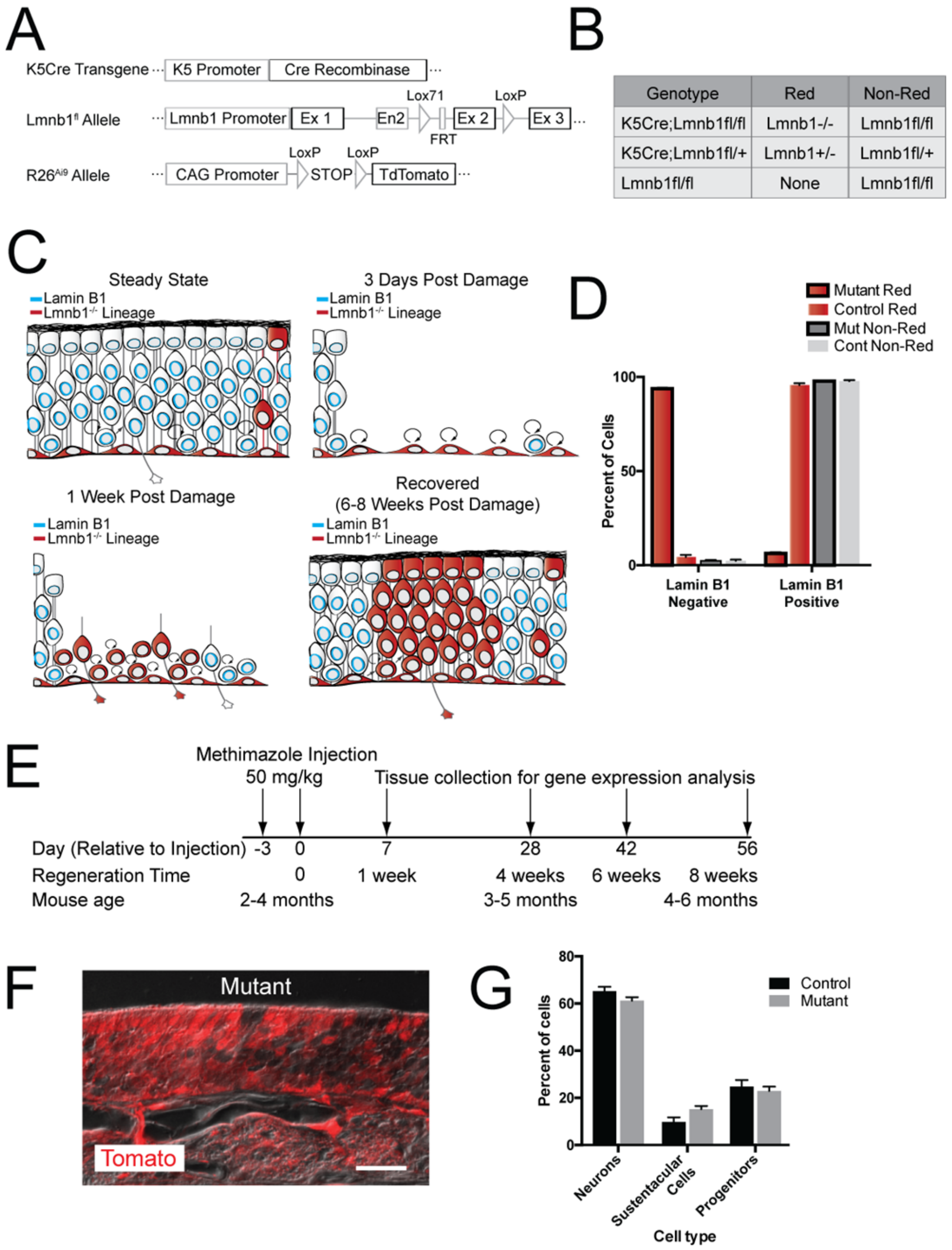

Supplementary Figure 1. Conditional ablation of *Lmnb1* in the olfactory epithelium

A. Diagram of alleles used in this study. K5: cytokeratin 5; R26: Rosa26 locus; En2 Engrailed 2 intronic sequence.

B. Expected genotypes of cells in Cre (horizontal basal cell, red) and non-Cre (other, non-red) lineages.

C. Cartoons depicting the predicted recovery of *Lmnb1* mosaic mutant olfactory epithelium in response to Methimazole-induced damage. Under normal laboratory conditions (Steady-state), the olfactory epithelium consists of mostly mature neurons, with a typical pseudostratified organization. After damage, large areas of the epithelium exhibit cell loss and stem and progenitor cells of the olfactory epithelium are activated, including *Lmnb1*$^{-/-}$ (red) horizontal basal cells. One week after damage, the olfactory epithelium consists of mostly progenitors and immature neurons, with many proliferating cells. 6 to 8 weeks after damage, the olfactory epithelium has been fully recovered, both from *Lmnb1*$^{-/-}$ (red) horizontal basal cells and other non-mutant stem and progenitor cells of the epithelium.

D. Quantification of lamin B1 expression by antibody staining in the mosaic mutant and control olfactory epithelium. Quantification was performed after tissue regeneration. Tomato positive cells (red) are expected to be *Lmnb1*$^{-/-}$ in mutant and *Lmnb1*$^{+/-}$ in control tissue. Tomato negative cells are expected to be *Lmnb1*$^{fl/fl}$ in mutant and *Lmnb1*$^{+/fl}$ in control tissue.

E. Diagram of experimental timeline. Two methimazole injections were delivered two days apart to induce tissue damage and regeneration. Tissue collection was performed at several time points.

F. DIC image of olfactory epithelium from *Lmnb1* mosaic mutant (*K5Cre;Lmnb1*$^{fl/fl}$) olfactory epithelium after damage-induced activation of horizontal basal cells. Presumptive *Lmnb1*$^{-/-}$ cells (horizontal basal cell lineage) express the Cre-dependent Tomato reporter. Scale bar indicates 25 µm.

G. Quantification of the tomato positive cell types produced by presumptive *Lmnb1*$^{-/-}$ (mutant) and *Lmnb1*$^{+/-}$ (control) horizontal basal cells after tissue regeneration.

Supplementary Figure 2

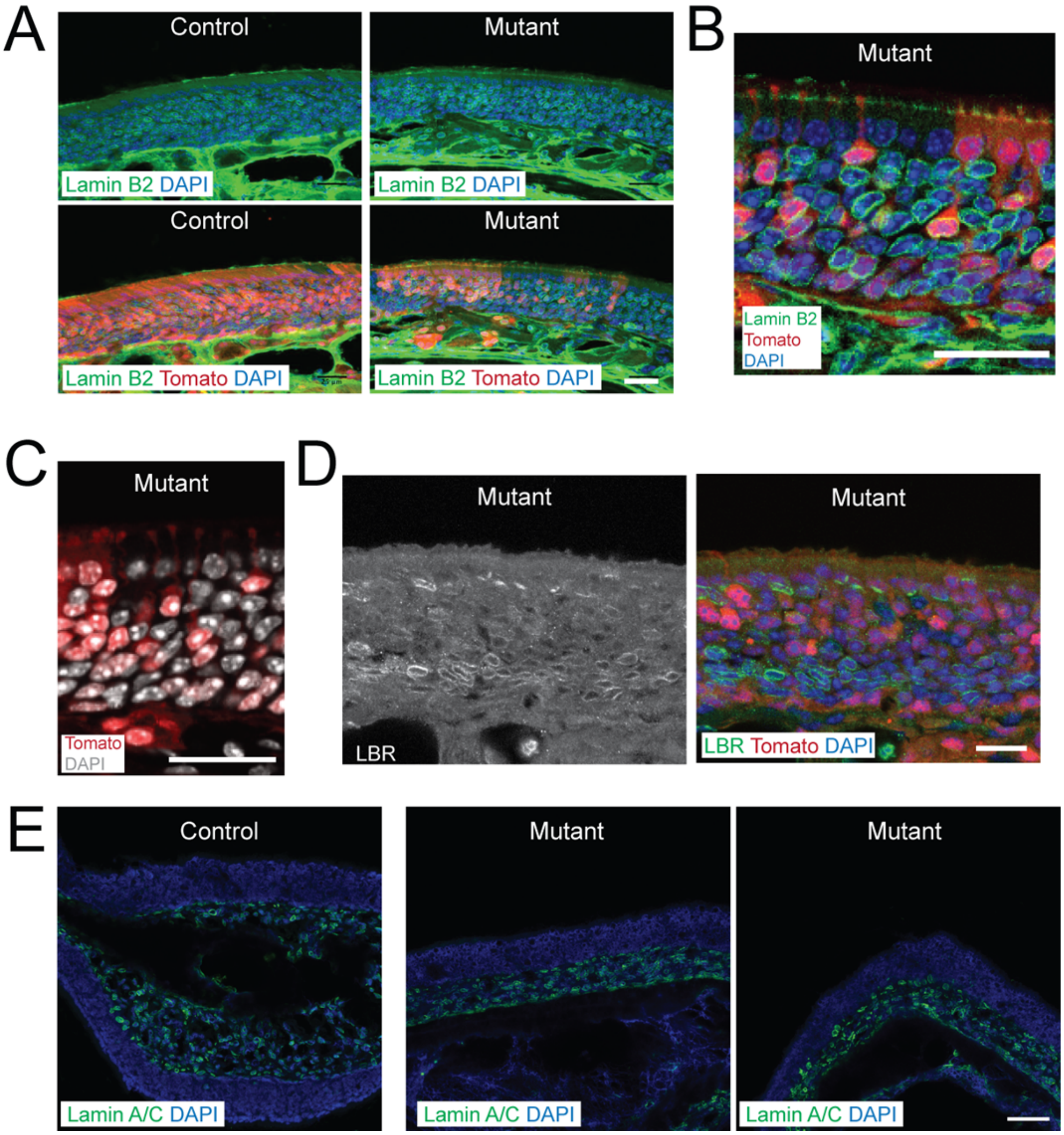

Supplementary Figure 2. Nuclear lamina organization in the *Lmnb1* mosaic mutant olfactory epithelium

A. Antibody staining for lamin B2 in control (*K5Cre;Lmnb1*$^{fl/+}$) and mosaic mutant (*K5Cre; Lmnb1*$^{fl/fl}$) olfactory epithelium sections. Tomato-positive cells in mutant are *Lmnb1*$^{-/-}$ in mosaic mutant, *Lmnb1*$^{+/-}$ in control. Scale bar indicates 25 µm.

B. Increased magnification showing lamin B2 staining in mosaic mutant olfactory epithelium. Tomato-positive cells are *Lmnb1*$^{-/-}$. Scale bar indicates 25 µm.

C. DAPI staining showing gross shape of nuclei in *Lmnb1* mosaic mutant epithelium. Tomato-positive cells are *Lmnb1*$^{-/-}$. Scale bar indicates 25 µm.

D. Antibody staining for Lamin B Receptor (LBR) in mosaic mutant olfactory epithelium. LBR is excluded from the neuronal cell layer but can be seen in the apical supporting cell and basal progenitor layers of the epithelium. Tomato-positive cells are *Lmnb1*$^{-/-}$. Scale bar indicates 25 µm.

E. Antibody staining for lamin A/C in mosaic mutant and control olfactory epithelium. Lamin A/C levels in the olfactory epithelium are below the detection limit for antibody staining, but cells in the basal lamina underlying the olfactory epithelium show positive staining. Scale bar indicates 50 µm.

Supplementary Figure 3

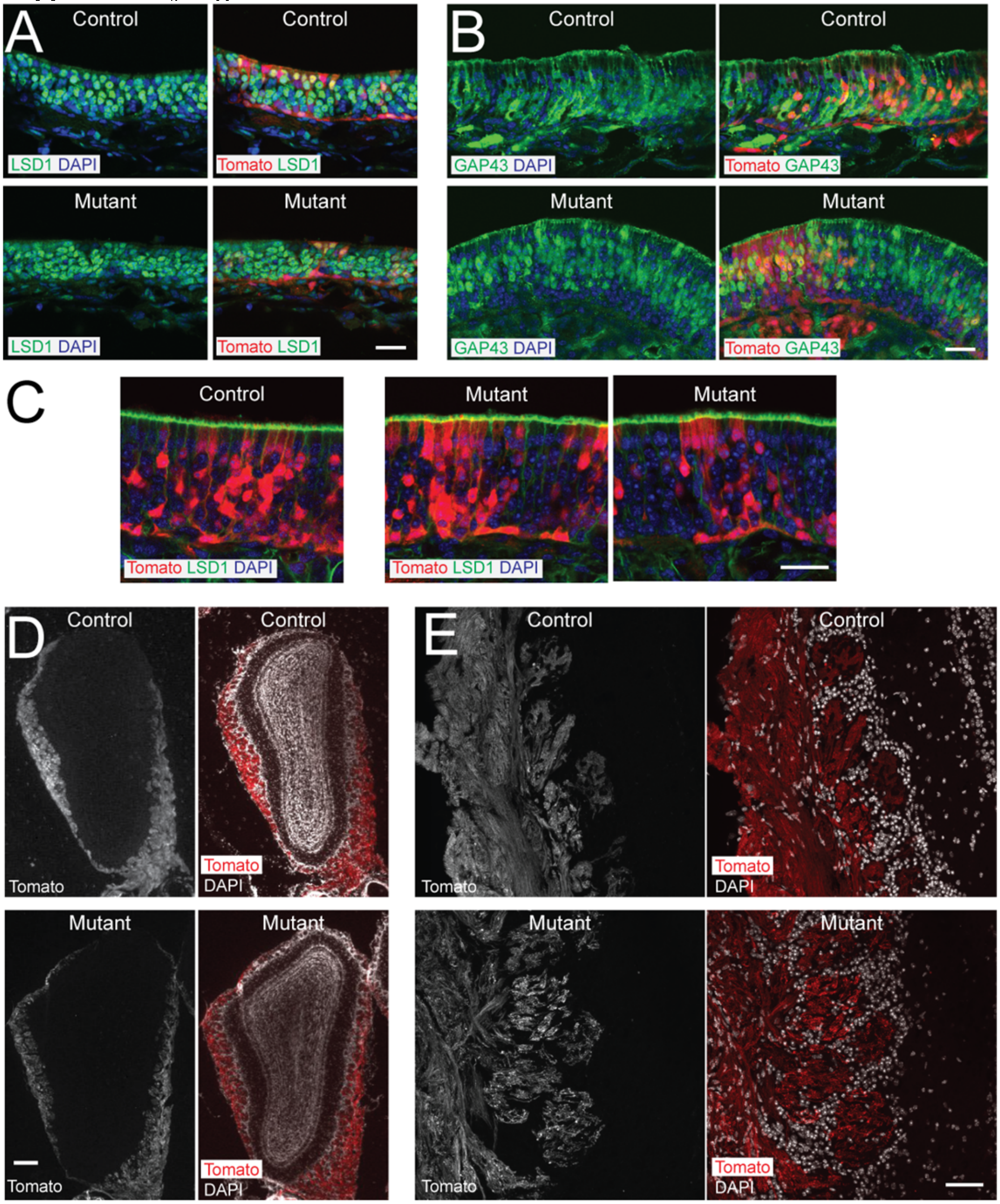

Supplementary Figure 3. Progenitor activation and axon targeting of *Lmnb1*$^{-/-}$ cells in *Lmnb1* mosaic mutant olfactory epithelia

A. Antibody staining of olfactory neuron progenitor marker LSD1 in mosaic mutant and control olfactory epithelium 1 week after regeneration. Horizontal basal cell lineage can be identified by Tomato expression. Scale bar indicates 25 µm.

B. Expression of immature neuron marker GAP43 during tissue recovery, at 3 weeks after damage-induced regeneration. Horizontal basal cell lineage can be identified by Tomato expression. Scale bar indicates 25 µm.

C. Phalloidin staining of sustentacular cell microvilli in mosaic mutant and control olfactory epithelium. Scale bar indicates 25 µm.

D. Innervation of the olfactory bulb by Tomato-positive olfactory sensory neuron axons from control (*Lmnb1*$^{+/-}$) and mutant (*Lmnb1*$^{-/-}$) olfactory neurons. DAPI staining shows nuclei in the olfactory bulb and neuropil glomerular regions innervated by olfactory sensory neurons.

E. Increased magnification of olfactory bulb innervation by *Lmnb1*$^{+/-}$ and *Lmnb1*$^{-/-}$ olfactory sensory neurons. Scale bar indicates 50 µm.

Supplementary Figure 4

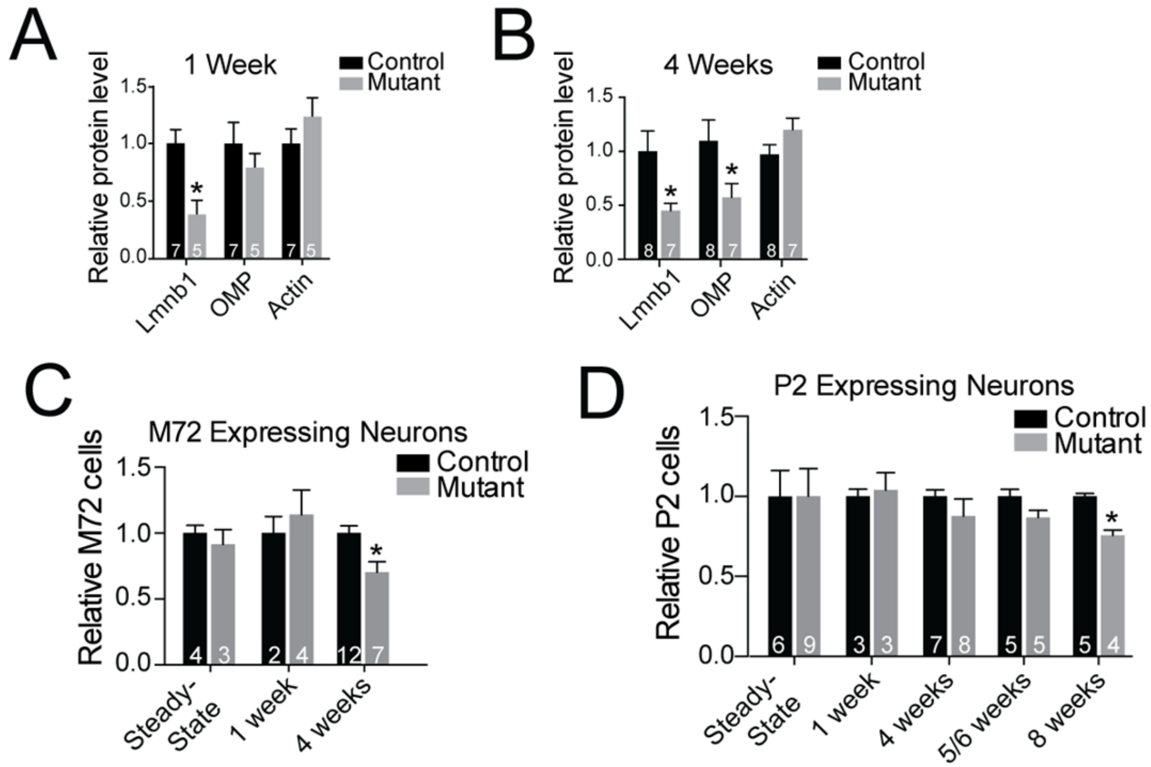

Supplementary Figure 4. Expression of mature olfactory sensory neuron proteins in the *Lmnb1* mosaic mutant olfactory epithelium

A–B. Quantification of Western data indicating protein levels in mosaic mutant and control olfactory mucosa samples from tissue 1 (A) or 4 (B) weeks after Methimazole-induced regeneration. All values (except beta actin) were normalized to beta actin for the same sample. All values are plotted relative to the average control. Data are expressed as mean + SEM. Number of samples (n) is shown on each bar; each sample was taken from one animal. * $p<0.05$ *unpaired student's t test, corrected for multiple comparisons using Holm-Sidak test.*

C–D. Quantification of expression of odorant receptor reporter alleles, *M72-IRES-tauLacZ* or *P2-IRES-tauLacZ*, in mosaic mutant and control olfactory epithelia. Data are plotted relative to the average value for controls in the same experimental group, for comparison across time points. All data are expressed as mean + SEM; n value is shown on each bar; each n represents data from one animal. *$p<0.05$ *unpaired Student's t test*.

Supplementary Figure 5

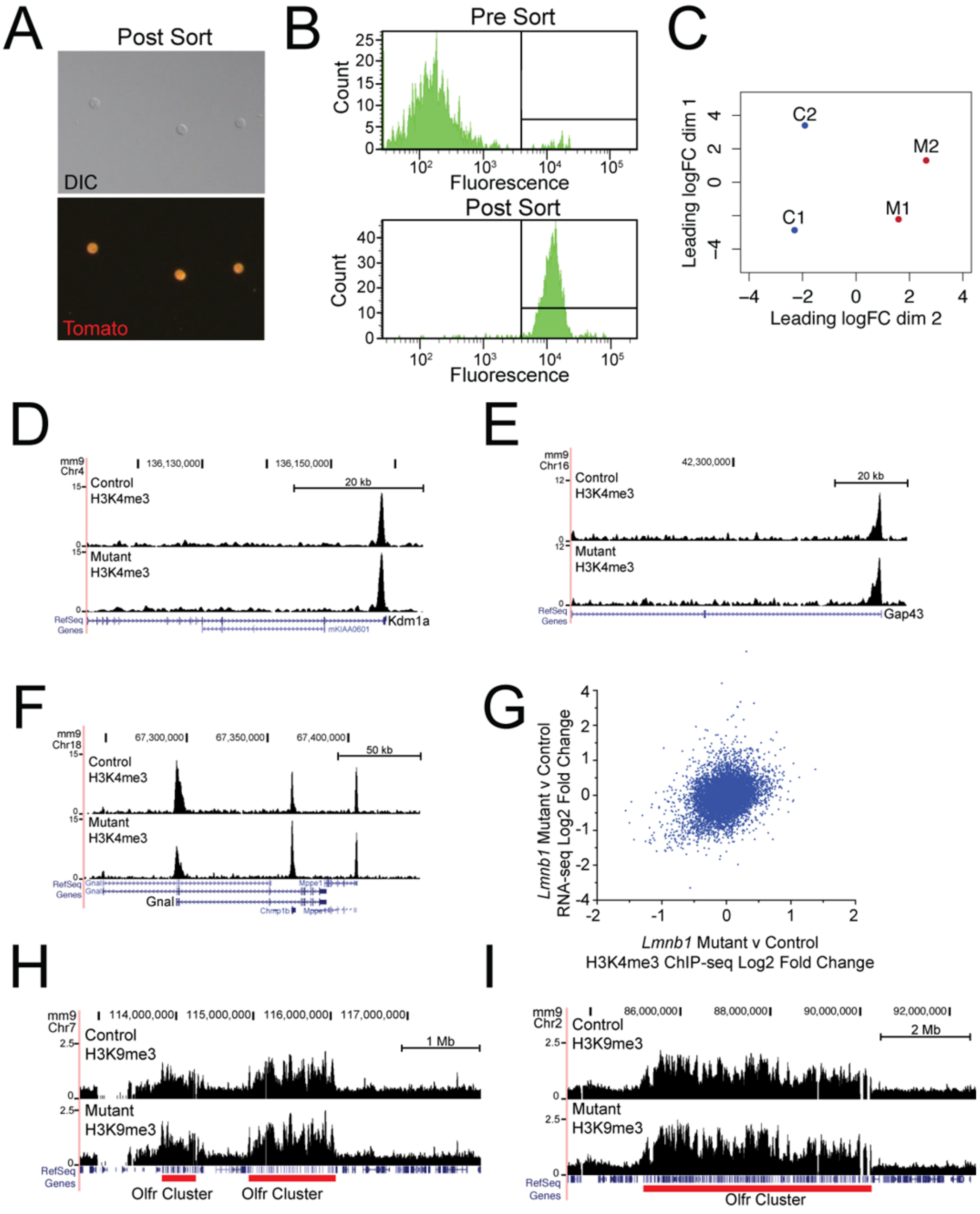

Supplementary Figure 5. Fluorescence-Activated Cell Sorting and epigenetic landscape of *Lmnb1*$^{-/-}$ cells

A. Enrichment for Tomato-expressing cells by Fluorescence-Activated Cell Sorting (FACS). Fluorescence and DIC images of dissociated *Lmnb1*$^{-/-}$ olfactory cells after fluorescence-activated cell sorting (FACS).

B. Enrichment for Tomato-expressing cells by FACS. Histograms showing the distribution of dissociated olfactory epithelium cells from mice expressing K5Cre and Tomato reporter allele 4 weeks after damage-induced regeneration. Counts and relative fluorescence are depicted before (top) and after (bottom) FACS.

C. MDS plot representing the differences between four samples, each from individual mice used in RNA-seq. M1 and C1 are samples from mutant and control littermates in group 1, M2 and C2 are mutant and control littermates from group 2. Dimension 2 highlights the differences between genotypes, while dimension 1 highlights the differences between sample groups (litter, batch, treatment).

D. Representative histogram showing H3K4me3 distribution at the promoter region of lysine specific demethylase 1 (LSD1, *Kdm1a*), typically expressed in olfactory neuron progenitors, based on FARP-ChIP-seq reads from sorted *Lmnb1*$^{-/-}$ and *Lmnb1*$^{+/-}$ cells.

E. Representative histogram showing H3K4me3 distribution at the promoter of immature neuron gene *Gap43*, based on FARP-ChIP-seq reads from mutant and control cells.

F. Representative histogram showing H3K4me3 distribution over mature neuron gene G_α signaling protein G_{olf} (*Gnal*), based on FARP-ChIP-seq.

G. Correlation between changes in gene expression (log2 fold change from RNA-seq analysis) and H3K4me3 promoter abundance (log2 fold change from FARP-ChIP-seq analysis) in mutant cells compared to controls for all genes analyzed. $R=0.29$, $P < 0.001$

G–H. Representative histograms showing H3K9me3 distribution over odorant receptor clusters on chromosomes 2 and 7 based on FARP-ChIP-seq.

Supplementary Figure 6

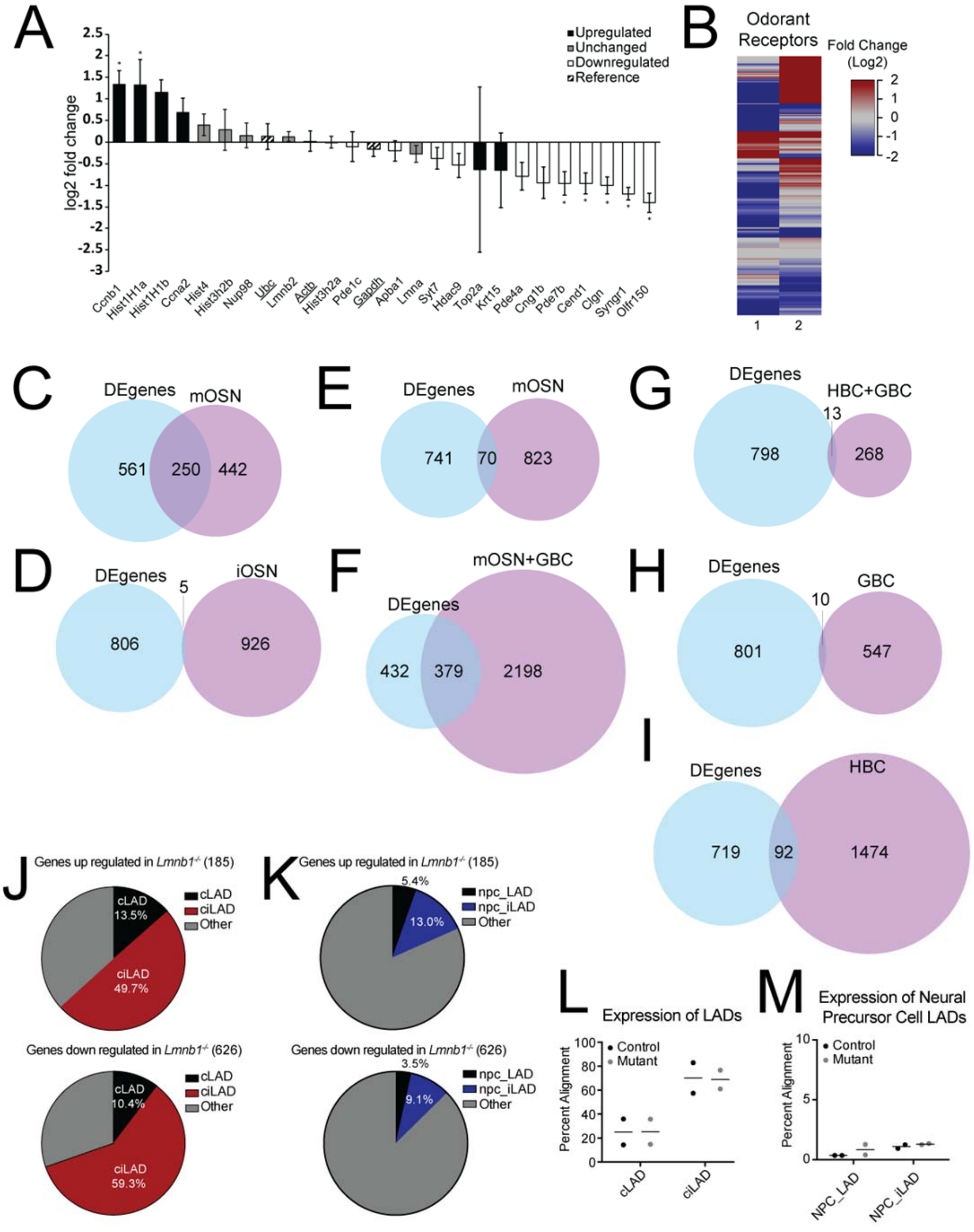

Supplementary Figure 6. Comparative analysis of RNA-seq data from sorted *Lmnb1*$^{-/-}$ cells

A. Bar graph showing fold change in expression of candidate genes in mosaic mutant compared to control olfactory epithelium four weeks after regeneration by qPCR. Bars are shaded according to the RNA-seq differential expression analysis for each gene. All data were normalized to the geometric mean of Actb (beta actin), Gapdh, and Ubc (ubiquitin C). Mean and SEM are shown. * *$p<0.05$ one-tailed t test*

B. Heatmap showing log2 fold change in gene expression between *Lmnb1*$^{-/-}$ and *Lmnb1*$^{+/-}$ controls for genes expressed in a subset of odorant receptor genes. Data is shown for two experimental replicates (1 and 2).

C–D. Venn diagrams showing the overlap between genes differentially expressed in *Lmnb1*$^{-/-}$ cells based on RNA-seq analysis (DE genes) and genes expressed in mature and immature olfactory sensory neurons based on a previous characterization[1].

E–I. Venn diagrams showing the overlap between genes differentially expressed in *Lmnb1*$^{-/-}$ cells based on RNA-seq analysis (DE genes) and genes expressed in mature olfactory neurons and different progenitor cell types based on a previous characterization[2].

J–K. Diagram showing proportion of genes differentially expressed in *Lmnb1*$^{-/-}$ cells that are found in common lamina-associated domains (cLAD), common inter LAD (ciLAD) or neural precursor lamina-associated domains (NPC-LAD), neural precursor inter LAD (NPC-iLAD). LAD sequences were identified in a previous study in cultured cells[3].

L–M. Expression level of genes found in lamina-associated domains in *Lmnb1*$^{-/-}$ and *Lmnb1*$^{+/-}$ cells. RNA-seq reads were aligned to either common LAD and common

interLAD sequences (M) or neural precursor LAD sequences or neural precursor interLAD sequences (N); LAD sequences were identified in a previous study in cultured cells[3]; percent alignment is shown.

Supplementary Figure 7

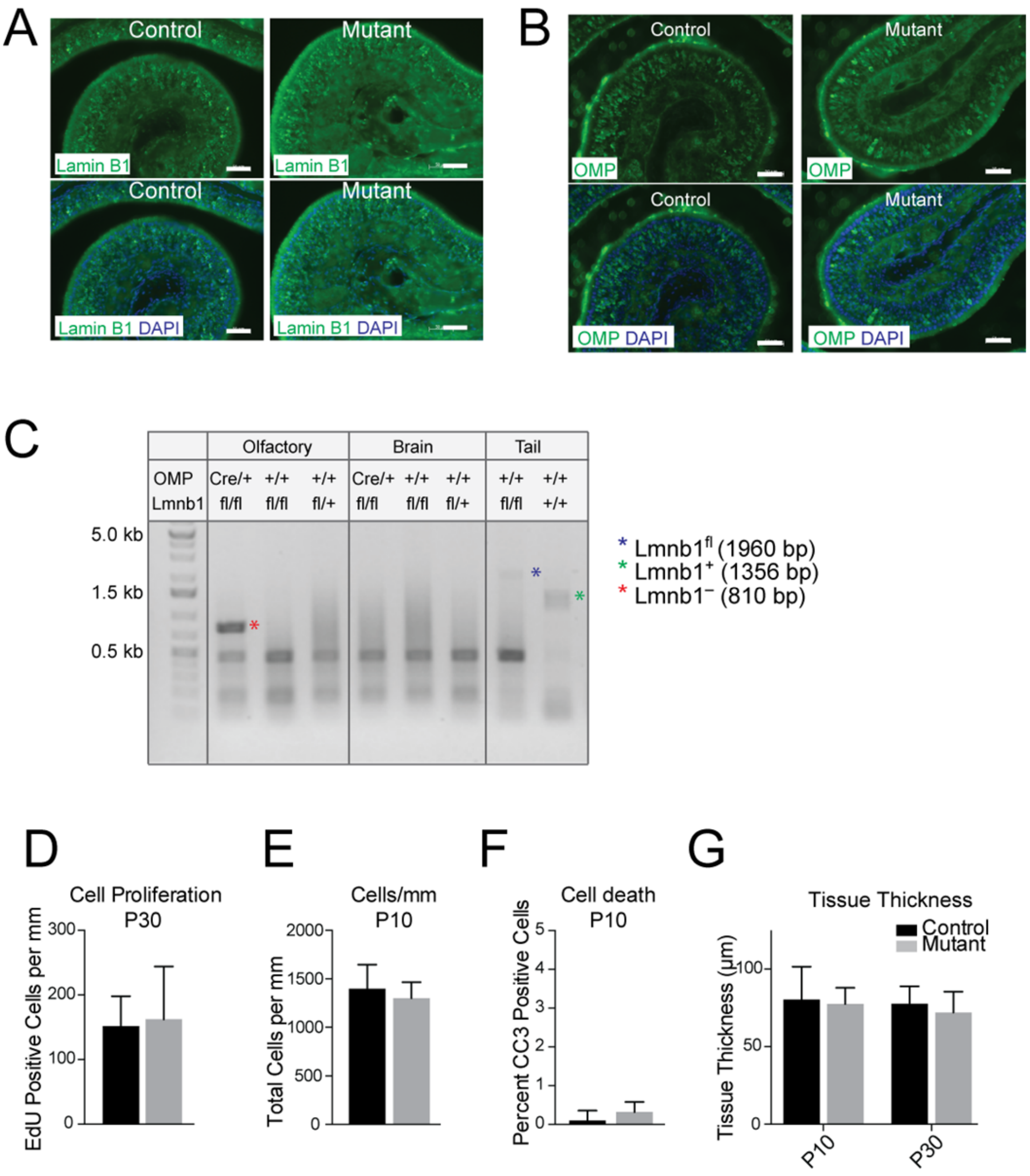

Supplementary Figure 7. *Lmnb1* conditional knockout in mature olfactory sensory neurons

A and B. Antibody staining for lamin B1 (A) and the mature olfactory neuron marker OMP (B) in olfactory epithelium from conditional mutant ($Omp^{Cre/+}$;$Lmnb1^{fl/fl}$) and control littermate ($Omp^{+/+}$;$Lmnb1^{fl/fl}$) mice. Scale bars indicate 50 µm.

C. PCR of genomic DNA isolated from olfactory mucosa (Olfactory), brain, or tail samples from *Lmnb1* conditional mutant and littermate control mice. Mouse genotype is given in rows marked OMP and Lmnb1. Amplification of $Lmnb1^{fl}$ allele, *Lmnb1* knockout allele (recombined $Lmnb1^{fl}$ allele, $Lmnb1^{-}$), and wildtype allele ($Lmnb1^{+}$) are shown by colored asterisks. Bands below 500 bp are non-specific. Amplification of the knockout allele was only ever observed in mutant samples from the olfactory epithelium.

D. Quantification of cell proliferation in the olfactory epithelium of conditional mutant and control littermate mice 30 days after birth (P30). Proliferation rate was determined by EdU retention after a 24-hour pulse.

E. Number of cells per millimeter of olfactory epithelium in the conditional mutant and the control at postnatal day 10 (P10).

F. Cell death, measured by antibody staining for cleaved caspase 3 (CC3), in conditional mutant and littermate control mice.

G. Tissue thickness of the olfactory epithelium in OMP-driven *Lmnb1* conditional mutant and littermate control mice at P10 and P30. For all bar graphs, mean is shown with SEM.

Supplementary Figure 8

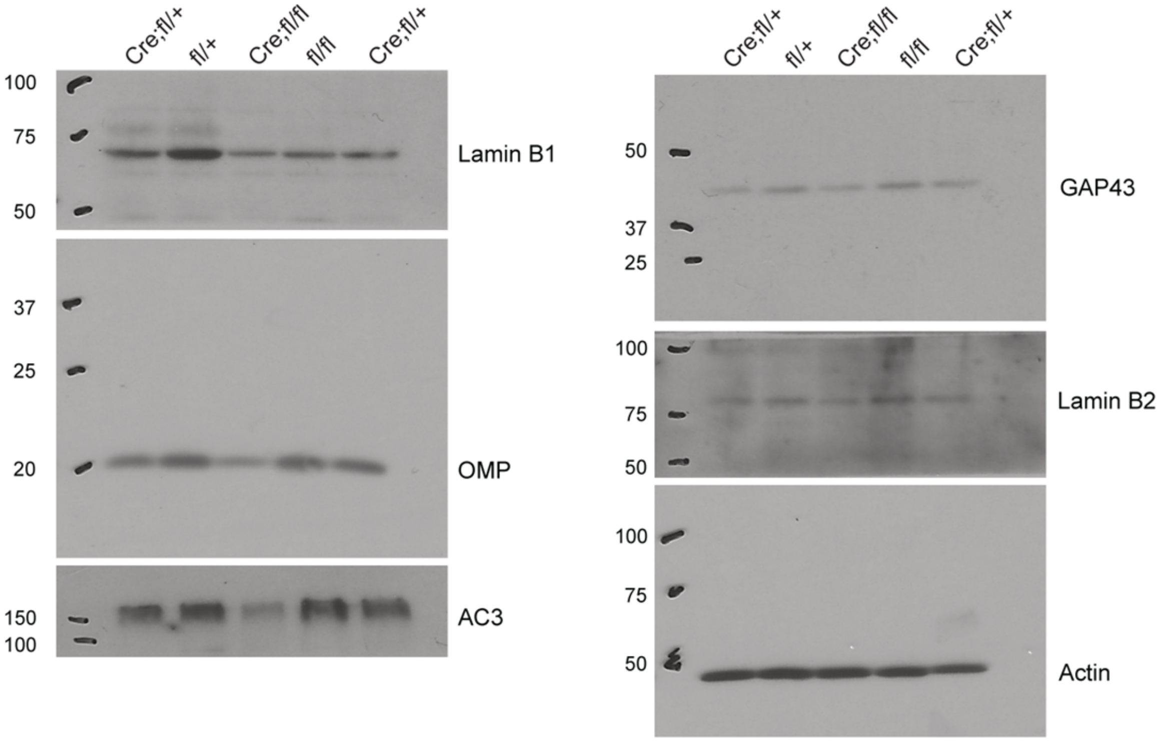

Supplementary Figure 8. Uncropped Western blot images.

Cropped versions of these images are shown in Figure 4C. Mouse genotypes are *K5Cre;Lmnb1*$^{fl/fl}$ (Cre;fl/fl), *Lmnb1*$^{fl/fl}$ (fl/fl) and *K5Cre;Lmnb1*$^{fl/+}$ (Cre;fl/+).

Supplementary Table 1. Primers used in this study

Gene	Forward Primer	Reverse Primer
Actb	GGCTGTATTCCCCTCCATCG	CCAGTTGGTAACAATGCCATGT
Abpa1	GGTGCTGAGTCATCAAGCATAC	GAACTTCAACGTAGGTTGGGAA
Ccna2	TGATGCTTGTCAAATGCTCAGC	AGGTCCTCCTGTACTGCTCAT
Ccnb1	AAGGTGCCTGTGTGTGAACC	GTCAGCCCCATCATCTGCG
Cend1	ACCAGCCAAGGCAGATCCT	GGTCAAGTTCTCACAAGGCCA
Clgn	CCAGGGTGTTGGACTATGTTTG	CCCCGAGGAAGGTTCATCTTTA
Cng1b	CAGAGGAGGAACACTACTGCG	AAGTAATCCATGAGGAGCCAGA
Gapdh	TGCACCACCAACTGCTTAGC	GGCATGGACTGTGGTCATGAG
Hdac9	GCGGTCCAGGTTAAAACAGAA	GCCACCTCAAACACTCGCTT
Hist1h1a	ACTGCCACGGAGAAACCTG	AGAAACTGCCTGCACGATGAG
Hist1h1b	CTCCTGTAGAGAAGTCTCCCG	GCAGAAACAGCCTTAGTGATGAG
Hist3h2a	AAGCTCGTGCAAAAGCGAAG	GCAGTCAGATATTCTAGCACAGC
Hist3h2b	TCCGCGTTTCCGTAGTACAAC	GGGGCTGGAGCTGATTTGG
Hist4	GAAGCGCATCTCGGGTCTC	CATAGCCGTAACCGTCTTGC
Krt15	AGCTATTGCAGAGAAAAACCGT	GGTCCGTCTCAGGTCTGTG
Lmna	ACCCCGCTGAGTACAACCT	TTCGAGTGACTGTGACACTGG
Lmnb2	TGGCATCAAGACCCTGTACGA	TCAGCCTCACTCCGGTGAAA
Nup98	GATTAGGGGGCTTTGGTACAAC	CTCCTGGTTTGGTCTGTGAATTT
Olfr536	ATGGAGGATGCCTCATACAGAT	ATGGAGGATGCCTCATACAGAT
Pde1c	GTCCCAGCGTCGTGATTAGC	TCATGACATCTCGAGCAAGTCTTT
Pde4a	GCCATGGAACAGTCAAAGGT	ATGTGCTGAGGCTGTCTCCT
Pde7b	AGCAGCTGGGCTCCCTCATCTT	CAGCACACTTCAAGGCGATCTGA
Syngr1	CCCAGGACTACATGGACCC	CAAAGGCGTTGGCTGGATG
Syt	CCGTCAGCCTTAGCGTCAC	GCAGGCAACTTGATGGCTTTC
Top2A	CAACTGGAACATATACTGCTCCG	GGGTCCCTTTGTTTGTTATCAGC
Ubc	GCCCAGTGTTACCACCAAGA	CCCATCACACCCAAGAACA

Supplementary Text

Supplementary Methods

RNA-seq

Transcriptome profiles were generated from sorted mutant and control (heterozygous) cells from regenerated olfactory epithelium. Each sample was collected from one mouse. Data are from two experimental groups, each containing a control and a mutant sample. Within a group, treatment, sample preparation, sequencing, animal sex, age, and parents were the same. Cells were sorted from two independent experimental groups of animals; within a group, mutant and control animals were littermates of the same sex and treatment group. 150,000 to 600,000 Tomato-positive mutant or heterozygous olfactory epithelial cells from a single mouse were sorted directly into TRIzol® solution (Life Technologies 15596026). RNA extraction was performed as described (TRIzol® protocol, Life Technologies). Genomic DNA was digested using RNase-free DNaseI (Qiagen 79254) then RNA was purified using RNeasy (Qiagen 74104). Libraries were prepared using Illumina RNA Prep Kit 2 following Ribo-Zero Gold depletion of rRNA (Illumina MRZG126). Libraries were analyzed on an Agilent Bioanalyzer 2100 and sequenced using an Illumina HiSeq2000 to produce approximately 50 million 50 bp single-end reads per sample.

Reads that aligned to rRNA reads (mm10) using Bowtie2 [4] were removed. Remaining reads were quality filtered using FASTQ quality filter parameters –q 28 –p 95. Filtered reads were aligned to mouse genome mm10 using Tophat[5] standard settings

(--no-covereage-search). Tophat output was converted to counts using HtSeq[6] using --mode=intersection-nonempty. Differential expression analysis was performed using edgeR (Bioconductor, see below)[7,8]. Only genes expressed at 1 cpm in two or more samples were used in differential expression analysis. GO term analysis was performed using DAVID [9]. RNA sequencing reads were aligned to published datasets [1-3] using Bowtie2[4]. Bedtools intersect was used to determine proportion of differentially expressed genes in LADs using parameters –f 0.5 –c for comparison with common LADs and inter LADs and parameters –f 0.01 –c for NPC LADs and inter LADs. Heatmaps were made using heatmap.2 (gplots) and RColorBrewer. MDS plot was made in RStudio using edgeR plotMDS.DGElist. Venn diagrams were made by comparing gene lists using the ‘comm’ command (Linux/Unix) and the VennDiagram package in RStudio.

Differential expression analysis using edgeR:

```
counts <- cbind(sample1[,2],sample2[,2],sample3[,2],sample4[,2])
colnames(counts)=c("C1","C2","M1","M2")
Geno <- c("C","C","M","M")
Treat <- c(1,2,1,2)
design <- model.matrix(~Geno+Treat,counts=counts)
x=DGEList(counts, group=Geno)
dim(x)
        [1] 23342     4
apply(x, 2, sum)
        wt1    wt2    mt1    mt2
        99198332 39169676 99249768 26089834
keep <- rowSums(cpm(x)>1) >= 2
x <- x [keep,]
dim(x)
        [1] 13560     4
apply(x, 2, sum)
        wt1    wt2    mt1    mt2
        99090228 39094399 99076671 26040737
x$samples$lib.size <- colSums(x$counts)
colSums(x$counts)
```

```
x<-calcNormFactors(x)
x<-estimateGLMCommonDisp(x, design)
x<-estimateGLMTrendedDisp(x, design)
x <- estimateGLMTagwiseDisp(x,design)
fit <- glmFit(x, design)
lrt <- glmLRT(fit,coef=2)
topTags(lrt)
de <- decideTestsDGE(lrt, p=0.05, adjust="BH")
summary(de)
        -1   626
         0 12749
         1   185
```

ChIP-seq

Samples were generated from sorted mutant and control (heterozygous) cells from regenerated olfactory epithelium from two experimental groups (littermate pairs of mutant and control animals). Within a group, treatment, sample preparation, sequencing, animal sex, and parents were the same. Each sample was collected from one mouse. Cells were sorted into sterile PBS and stored at −80°C until use. FARP-ChIP-seq was performed as described for low cell number[10]. Briefly, each sample (500,000 Tomato fluorescent cells from one animal) was split and incubated with either anti-H3K9me3 or anti-H3K4me3 overnight 4°C. Bound DNA was recovered following Millipore ChIP Assay kit protocol (17-295). Bacterial DNA and Biotinylated-DNA coupled Streptavidin beads (Invitrogen M280) were used as carriers as described[10]. Library preparation was performed according to Illumina TruSeq® DNA Sample Preparation Guide (15026486) with 12 (H3K4me3) or 14 (H3K9me3) cycles of PCR amplification. DNA libraries were analyzed on an Agilent Bioanalyzer 2100 before sequencing on an Illumina HiSeq2000 to produce approximately 50 million 50 bp single-end reads per sample.

ChIP-seq data analysis pipeline was performed as described[10]. More specifically, ChIP-seq reads were mapped to the mouse genome (mm9) using bowtie with parameters "-m 1 -v 2". Then ChIP-seq peaks were called using MACS [11] for H3K4me3 with parameters "--llocal=100000 -p 1.0e-5" or SICER [12] for H3K9me3 with E-value threshold 0.01. The peaks for control (*Lmnb1*$^{+/-}$) and mutant (*Lmnb1*$^{-/-}$) were then merged to form an overall peak set (for H3K4me3 and H3K9me3 individually). The read numbers that fall on each peak were counted using a custom script. The comparison between control and *Lmnb1*$^{-/-}$ was done by edgeR[7]. Specifically, the dispersion was set at 0.01 when calling differential peak usage.

qPCR

Following transcardial perfusion with PBS of each mouse, olfactory mucosa were dissected out and homogenized in TRIzol® (Life Technologies), and RNA was extracted according to the manufacturer's protocol. DNase I digestion of genomic DNA and subsequent RNA purification were performed with the RNeasy Plus Mini kit (Qiagen), according to the manufacturer's protocol. cDNA was generated from 1µg of RNA, as quantified by a Nanodrop 1000, using the RETROscript® Reverse Transcription Kit (Thermo Fisher Scientific) with Oligo dTs as primers and a reverse transcription temperature of 42˚C for 1 hour. qPCR reactions were performed on a StepOnePlus™ Real-Time PCR system (Applied Biosystems) using Maxima SYBR green/ROX qPCR Master Mix 2x (Thermo Fisher Scientific), with reactions formulated according to manufacturer recommendations. If two of the Ct values were within 0.5 of each other,

and the third Ct value was not within 0.5 of either of the other two, this Ct value was excluded from the analysis. Primer sequences can be found in Supplementary Table 1.

Cycling conditions were as follows: 1 cycle of 95˚C for 10 minutes; and 40 cycles of 95˚C for 15s, 60˚C for 30s, and 72˚C for 30s. All reactions were performed in triplicate, and Ct values were averaged for each gene in each sample. These results were analyzed by the 2 –ΔΔCt method[13] with normalization to the geometric mean of Actb, Gapdh, and Ubc[14]. Results are reported as log 2 of the fold change, equivalent to –ΔΔCt values. Each sample (n) was taken from one animal; littermates from the same treatment group were used as controls.

Supplementary References

1 Nickell, M. D., Breheny, P., Stromberg, A. J. & McClintock, T. S. Genomics of mature and immature olfactory sensory neurons. *The Journal of comparative neurology* **520**, 2608-2629, doi:10.1002/cne.23052 (2012).

2 Colquitt, B. M., Allen, W. E., Barnea, G. & Lomvardas, S. Alteration of genic 5-hydroxymethylcytosine patterning in olfactory neurons correlates with changes in gene expression and cell identity. *Proceedings of the National Academy of Sciences of the United States of America* **110**, 14682-14687, doi:10.1073/pnas.1302759110 (2013).

3 Peric-Hupkes, D. *et al.* Molecular maps of the reorganization of genome-nuclear lamina interactions during differentiation. *Molecular cell* **38**, 603-613, doi:10.1016/j.molcel.2010.03.016 (2010).

4 Langmead, B. & Salzberg, S. L. Fast gapped-read alignment with Bowtie 2. *Nature methods* **9**, 357-359, doi:10.1038/nmeth.1923 (2012).

5 Kim, D. *et al.* TopHat2: accurate alignment of transcriptomes in the presence of insertions, deletions and gene fusions. *Genome biology* **14**, R36, doi:10.1186/gb-2013-14-4-r36 (2013).

6 Anders, S., Pyl, P. T. & Huber, W. HTSeq--a Python framework to work with high-throughput sequencing data. *Bioinformatics* **31**, 166-169, doi:10.1093/bioinformatics/btu638 (2015).

7 Robinson, M. D., McCarthy, D. J. & Smyth, G. K. edgeR: a Bioconductor package for differential expression analysis of digital gene expression data. *Bioinformatics* **26**, 139-140, doi:10.1093/bioinformatics/btp616 (2010).

8 Zhou, X., Lindsay, H. & Robinson, M. D. Robustly detecting differential expression in RNA sequencing data using observation weights. *Nucleic acids research* **42**, e91, doi:10.1093/nar/gku310 (2014).

9 Huang da, W., Sherman, B. T. & Lempicki, R. A. Systematic and integrative analysis of large gene lists using DAVID bioinformatics resources. *Nature protocols* **4**, 44-57, doi:10.1038/nprot.2008.211 (2009).

10 Zheng, X. *et al.* Low-Cell-Number Epigenome Profiling Aids the Study of Lens Aging and Hematopoiesis. *Cell reports* **13**, 1505-1518, doi:10.1016/j.celrep.2015.10.004 (2015).

11 Zhang, Y. *et al.* Model-based analysis of ChIP-Seq (MACS). *Genome biology* **9**, R137, doi:10.1186/gb-2008-9-9-r137 (2008).

12 Zang, C. *et al.* A clustering approach for identification of enriched domains from histone modification ChIP-Seq data. *Bioinformatics* **25**, 1952-1958, doi:10.1093/bioinformatics/btp340 (2009).

13 Livak, K. J. & Schmittgen, T. D. Analysis of relative gene expression data using real-time quantitative PCR and the 2(-Delta Delta C(T)) Method. *Methods* **25**, 402-408, doi:10.1006/meth.2001.1262 (2001).

14 Vandesompele, J. *et al.* Accurate normalization of real-time quantitative RT-PCR data by geometric averaging of multiple internal control genes. *Genome biology* **3**, RESEARCH0034 (2002).
